# Response to Vaccination Against SARS-CoV-2 in Patients With Antineutrophil Cytoplasmic Antibody-Associated Vasculitis With Renal Involvement

**DOI:** 10.3389/fmed.2021.817845

**Published:** 2022-01-20

**Authors:** Jack E. Carruthers, James Wells, Arun Gupta, Delordson Kallon, Amber Cox, Neuza Pina, Muhammad Magdi Yaqoob, Ravindra Rajakariar

**Affiliations:** ^1^Department of Renal Medicine and Transplantation, Royal London Hospital, Barts Health NHS Trust, London, United Kingdom; ^2^The Francis Crick Institute, University College London, London, United Kingdom; ^3^Queen Mary University of London, London, United Kingdom

**Keywords:** COVID-19, ANCA-associated vasculitis, renal disease, seroconversion, public health, patient care

## Abstract

**Background:**

Patients with anti-neutrophil cytoplasmic antibody (ANCA)-associated vasculitides (AAV) present with multisystem disease including renal impairment. The treatment for AAV involves a high burden of immunosuppression. Patients with renal involvement are treated especially intensively. As a result, we identified these patients as being potentially at high risk of failure to seroconvert to COVID-19 vaccination.

**Methods:**

We collected data on seroconversion response rates to COVID-19 vaccination in a multi-ethnic cohort of patients with AAV and renal involvement treated at a busy tertiary nephrology centre as part of a retrospective review of patient notes. Blood samples were taken following vaccination with either Pfizer or Astra-Zeneca COVID-19 vaccines and median fluorescence intensity was measured using the validated MULTICOV-Ab Magnetic Luminex^Ⓡ^ Assay. We also evaluated whether seroconversion was affected by immunosuppression regimen.

**Results:**

81 patients were included. The mean age was 62, and there were 49 (60%) females. 55 patients had a blood test after the first dose; 46 after the second dose. Patients were in remission with a median BVAS of 0 (IQR 2). Seroconversion after the first dose with either vaccine was 35/55 (63.6%). After the second it was 38/46 (82.6%). Subgroup analyses revealed a trend to impaired seroconversion in non-white versus white patients (77.8 vs. 81.7% (*p* = 0.69) after the first dose of vaccine and in those treated with Rituximab in the last 12 months (73.3 vs. 87.1%, *p* = 0.41).

**Conclusions:**

These data offer real-world evidence of lower seroconversion in response to vaccination with one dose in patients with AAV and renal involvement than the general UK population. After two doses, seroconversion is in line with national data. These data provide a rationale for hospital-led identification of patients most at risk of COVID-19 and underscore the importance of local connexions between hospitals and their communities. These data provide further support for targeting booster vaccination programmes to vulnerable patient cohorts. They add to the growing evidence of reduced seroconversion in response to vaccination in patients with renal disease of any cause.

## Introduction

Anti-neutrophil cytoplasmic antibody (ANCA)-associated vasculitides (AAV) are a group of disorders including granulomatosis with polyangiitis (GPA), microscopic polyangiitis (MPA) and eosinophilic granulomatosis with polyangiitis (EGPA), characterised by multisystem inflammation. The pathogenesis is related to autoantibodies directed at autoantigens in the neutrophil cytoplasm. Estimates of the prevalence of AAV range from 30 to 218 per million in the population. The clinical consequences of AAV if untreated include respiratory and renal failure. Immunosuppression forms the basis of treatment, with induction immunosuppression through potent immunomodulatory agents such as cyclophosphamide, Rituximab and mycophenolate mofetil (MMF), usually combined with high-dose corticosteroids. The induction phase is often followed by long-term maintenance immunosuppression either with azathioprine, MMF, Rituximab or low-dose corticosteroids, often in combination (1). Patients with AAV and renal involvement are a group with particularly high burdens of immunosuppression and consequently high contact with tertiary referral centres.

Patients, including those with AAV and renal involvement, receiving such immunosuppressant therapies were excluded from the initial trials of COVID-19 vaccination, despite being at higher risk of poor outcomes from COVID-19 infection. In the UK, the national vaccination programme against COVID-19 has offered adults, including those with underlying autoimmune conditions, two doses of either the Pfizer-BioNTech (BNT162b2; hereafter, Pfizer) or Astra-Zeneca (ChAdOx1-S [recombinant]; AZ) vaccines since 8^th^ December 2020. At the time of writing, a third booster dose of vaccination was being offered to all vulnerable patients and patients aged over 55 years. Both randomised controlled trials of healthy participants from international populations and real-world data from the general UK population have revealed effective seroconversion in response to two doses of either vaccine, with estimates ranging from 75–95% (2–4).

Since then, descriptive studies have emerged assessing the serological response of specific patient groups to vaccination. These have revealed impaired seroconversion rates compared to the general population in patients living with end-stage renal failure (including those on dialysis) (5), solid-organ transplants (6) and in patients receiving immunosuppression as treatment for rheumatological diseases including AAV or cancer (7, 8).

Whether lower seroconversion rates to COVID-19 vaccination are due to the underlying disease or to the treatment for the disease is difficult to untangle. Rituximab is a monoclonal antibody against CD20, a B-cell antigen, and as such is considered a B-cell depleting therapy. Consequently, it may be implicated in reducing the humoral response to vaccination (9). As a result, it has come under much scrutiny in seroconversion studies. A recent report examining the effects of Rituximab on seroconversion after vaccination in a cohort of patients with diverse rheumatological diseases including rheumatoid arthritis and vasculitis found that Rituximab treatment per se was a negative indicator for seroconversion irrespective of the underlying disease (10). Other immunosuppressant therapies including methotrexate have also been revealed to affect seroconversion rates (11).

These studies have provided a rationale for targeting national booster vaccination programmes to vulnerable cohorts of patients including those with AAV. However, these studies do not provide a real-world view on seroconversion in patients with AAV. Moreover, there is no published data looking specifically at patients with AAV and renal involvement, a subgroup of patients who have particularly high immunosuppression burdens and require intensive hospital follow-up. These latter observations are important because hospitals with large cohorts of patients with AAV and renal involvement are likely to need to provide a bespoke blueprint for patient care in these cohorts to ensure they are protected from COVID-19 as much as is feasible.

To that end, we describe data collected from a multi-ethnic cohort of patients with AAV and renal involvement, under the care of a busy tertiary nephrology referral centre, who have been vaccinated against COVID-19. Assessment of serological response to vaccination has become a routine part of our clinical care, with the hope that this will be able to inform service provision and clinical decision making. We have audited this data and here present an overview of the response to two doses of COVID-19 vaccination in these patients. We provide the rationale for tailoring COVID-19 risk assessments at the hospital-level to ensure that these patients benefit from best care.

## Materials and Methods

### Data Collection

We examined the records of a cohort of patients under treatment and follow-up for AAV with renal involvement from any cause at a busy tertiary referral centre in London, UK. Patient identifiable features were anonymised and baseline demographic data including age, sex and ethnicity were collected. We collected data on their vasculitis diagnosis, their current Birmingham Vasculitis Activity Score (BVAS), the degree of renal impairment including whether they were on renal replacement therapy, the current estimated glomerular filtration rate (eGFR) if they were not on dialysis, and any other organ involvement they had sustained due to their diagnosis in addition to renal involvement. We recorded whether they had had a polymerase chain reaction (PCR)-positive COVID-19 infection at any point in the past. We recorded features of their immunosuppression regimens, separating data into “historical” and “current” immunosuppression. For example, a patient's historical immunosuppression may reflect induction with Rituximab and corticosteroids, whilst their current regimen is maintenance with azathioprine and corticosteroids. In this way, we were able to generate count data for historical vs. current exposure to steroids, Rituximab, azathioprine, cyclophosphamide and other immunosuppressants. For Rituximab, given the usual intervals between doses and the fact that the COVID-19 pandemic had affected the delivery of Rituximab to some of our patients, we set a cut-off of a dose prior to 12 months before vaccination as “historical” and any dose less than 12 months before vaccination as “current”.

Patients who had undertaken their first or second dose vaccinations with either Pfizer or AZ vaccines under the national vaccination programme in the UK were contacted in the period of time from December 2020 to April 2021. Patients consented to undertake a blood test under the care of the hospital phlebotomy service some 14–28 days after each dose. Some patients had a blood test only after their first dose, others only after their second dose, whilst some patients had two blood tests, after their first dose and after their second dose. This was an unavoidable reality of coordinating blood tests during the pandemic. For the purposes of analysis, all blood tests taken after the first dose were treated as one group and vice versa.

### Laboratory Analysis

Samples were tested using the validated MULTICOV-Ab Magnetic Luminex^Ⓡ^ Assay for detection of antibody responses to SARS-CoV 2 antigens (12, 13). This assay detects human IgG antibodies to six specific SARS-CoV 2 antigens, including the spike protein (S-protein) trimer, S-protein domain 1 (S1), S-protein domain 2 (S2), the receptor binding domain (RBD) of the S-protein, the N-terminal domain of nucleocapsid (N-NTD) and the nucleocapsid (14). The rationale for using the MULTICOV-Ab test as opposed to other commercially available tests was that its assay of multiple SARS-CoV 2 antigens improves sensitivity and specificity in detecting seroconversion responses to COVID-19 when compared to tests of just one S-protein antigen (13). In brief, patient samples are incubated with a panel of beads coated in the above SARS-CoV 2 antigens. The beads are labelled with fluorescent R-phycoerythrin-conjugated anti-human IgG. The sample is run through a Luminex fluorescence-activated cell sorter (FACS), generating a median fluorescence intensity (MFI) for each bead. This value is normalised to the fluorescence from a quality control sample allowing for cut-off values of >1 for RBD and S-protein positivity and >0.2 for nucleocapsid positivity.

Patients positive for RBD and/or the S-protein antigens were judged to have effectively seroconverted to vaccine whilst patients with positivity to RBD, S-protein and nucleocapsid were judged to have seroconverted to a combination of previous infection with COVID-19 and vaccination. Thus, the test also allowed us to retrospectively identify any patients who may have had COVID-19 infection that were not picked up by conventional testing.

We analysed seroconversion positivity rates for patients after first and second dose of vaccination from any vaccine. We also assessed seroconversion rates by vaccine type, either Pfizer or AZ. Finally, we examined their health records to review seroconversion rates by immunosuppression regimen as outlined above.

### Statistical Analysis

The primary goal was to evaluate percentage positivity following vaccination. For 2 by 2 contingency tables, we undertook Fisher's exact test. For continuous data, we undertook Mann-Whitney tests (significance *p* < 0.05). We used GraphPad Prism 9 (California, USA) software, unless otherwise stated in the text.

## Results

### Demographic Data

The records of 81 patients with AAV were identified and screened in the period of time. Patients had been diagnosed any time in the last 10 years. The mean age of the cohort was 62, and there were 49 (60%) females. Fifty five patients had had a blood test after their first dose whilst 46 patients had had a blood test after their second dose at the time of writing. A total of 20 patients had two blood tests, one after their first dose and one after their second dose.

Of the 81 patients, 41 had a diagnosis of GPA with proteinase 3 (PR3) antibody positivity and 36 MPA with myeloperoxidase (MPO) antibody positivity. The remaining four patients met clinical criteria for EGPA. All patients had renal involvement. Median creatinine for the whole group was 121.5 (IQR 89.8); median eGFR was 41.5 (IQR 32.75) excluding those on dialysis. Nine patients were on dialysis; two were transplanted. Additional organ involvement is shown in Table 1. All patients were in remission with median BVAS of 0 (IQR 2).

**Table 1:** Patient characteristics at baseline.

	**Granulomatosis**	**Microscopic**	**Eosinophilic**
	**with polyangiitis**	**polyangiitis**	**granulomatosis**
	**(GPA) (*n =* 41)**	**(MPA) (*n =* 36)**	**with polyangiitis**
			**(*n =* 4)**
Age (mean years)	60.7	60.0	57.5
Male sex [count (%)]	15 (36.6)	16 (44.4)	1 (25)
Kidney function
Median Cr (IQR)	108 (65)	156 (99)	125 (43)
Median eGFR (IQR)	50.5 (35.5)	34.5 (26.3)	47 (23.5)
Undergoing dialysis [count (%)]	4 (9.7)	5 (13.8)	0 (0)
Transplanted [count (%)]	2 (4.8)	0 (0)	0 (0)
Median BVAS (IQR)	0 (0.5)	2 (3.25)	1 (2)
**Organ involvement in whole cohort (*****n** **=*** **81) [count (%)]**			
Respiratory	13 (16)	
Mucous membranes including eyes	4 (4.9)	
Cutaneous	2 (2.5)	
Ear, nose, throat	8 (9.9)	
Neurological	6 (7.4)	
Renal	81 (100)	

### Primary Analysis

After the first dose with either vaccine, 35/55 (63.6%) had positive IgG antibodies indicating seroconversion whilst 20/55 (36.4%) were negative. After a second dose with either vaccine, 38/46 (82.6%) patients were positive for IgG antibodies to vaccination, whilst 8 patients were negative (17.4%). Three of these patients had had two blood tests. Seventeen patients had a PCR swab-positive COVID-19 infection prior to vaccination. All of the patients with a previous PCR positive COVID-19 swab seroconverted, 12 after one vaccine dose and 5 after two vaccine doses. No patients were identified as having had prior infection that had not previously been PCR-swab positive. Only one patient developed swab-positive COVID-19 infection after two doses of vaccine in the study period.

Median fluorescence intensity (MFI) after the first and second doses can be visualised in Table 2. Average MFI for S-protein antibodies after the first and second dose was 8,358 and 12,442 respectively (median 3,409 and 12,128). Average MFI for RBD antibodies was 7,068 and 9,526 (median 1,110 and 4,629) after first and second dose respectively.

**Table 2:** MFI by vaccine dose (both vaccines).

	**First dose**	**Second dose**	**Significance**
Mean MFI spike	8,358	12,442	0.70
Median MFI spike	3,409	12,128	
Mean MFI RBD	7,068	9,526	0.18
Median MFI RBD	1,110	4,629	

### Subgroup Analyses

The mean age of those who seroconverted after two doses of vaccine was 62.6 years, compared to 56.0 years amongst those who did not seroconvert. 17/20 (85.0%) of males responded, compared to 21/26 (80.8%) of females. Our population group is highly diverse and so we were interested in seroconversion rates by ethnicity. Seroconversion was seen in 24/28 (85.7%) of white patients, compared to 14/18 (77.8%) of non-white patients after one dose (Table 3). Equal rates of seroconversion were seen in white and non-white patients after two doses.

**Table 3:** Factors influencing seroconversion rates after 2 vaccine doses.

	**Positive**	**Negative**	**Significance**
	**seroconversion**	**seroconversion**	
Age (mean years)	62.6	56.0	
Sex			>0.99 (M vs. F)
Male	17/20 (85%)	3/20 (15%)	
Female	21/26 (81%)	5/26 (19%)	
Ethnicity			0.69 (White vs. non-white)
White	24/28 (86%)	4/28 (14%)	
South Asian	10/13 (77%)	3/13 (23%)	
All non-white	14/18 (77%)	4/18 (23%)	
Vaccination type			0.44 (AZ vs. Pfizer)
AZ	25/29 (86%)	4/29 (14%)	
Pfizer	13/17 (76%)	4/17 (24%)	
Previous infection		
Known COVID infection (seroconversion after one or two doses)	17	0	

We wanted to understand whether vaccination type affected seroconversion rates. 36/54 (66.7%) patients had received a first dose of the AZ vaccine (one unknown vaccine type), with 26/36 (72.2%) seroconverting. Eighteen patients had received a first dose of the Pfizer vaccine with 9/18 (50.0%) seroconverting. After the second dose, 25/29 (86.2%) of patients seroconverted with AZ, whilst 13/17 (76.5%) seroconverted with Pfizer (Table 2).

We wanted to understand whether immunosuppression affected seroconversion rates (Table 4). Our patients were induced with either cyclophosphamide or Rituximab and maintained with steroids, azathioprine, or MMF in line with local treatment expertise. All patients were in remission prior to their vaccination. Eight patients had received historic treatment with rituximab (more than 12 months prior to vaccination), of whom 7 (87.5%) seroconverted in response to two vaccine doses. Twenty nine patients had previously been treated with cyclophosphamide and 24/29 (82.8%) of this group responded positively to two doses of vaccination.

**Table 4:** Analysis of seroconversion by immunosuppressive regimen.

	**Seroconversion in**	**Seroconversion in**	**Significance**
	**those exposed**	**those unexposed**	
	**to treatment**	**to treatment**	
Rituximab (>12M before vaccine)	7/8 (87.5%)	20/23 (87.0%)*	>0.99
Rituximab (Within last 12M)	11/15 (73.3%)	27/31 (87.1%)	0.41
Cyclophosphamide (Ever)	24/29 (82.8%)	14/17 (82.6%)	>0.99
MMF (Current)	6/7 (85.7%)	32/39 (82.1%)	>0.99
AZA (Current)	14/16 (87.5%)	24/30 (80.0%)	0.69
Steroid (Current)	21/27 (77.8%)	17/19 (89.5%)	0.44

* *Figure excludes those who received Rituximab within the last 12 months*.

For current maintenance therapy, seroconversion rates were 6/7 (85.7%) for those currently taking MMF, 14/16 (87.5%) for those currently taking azathioprine and 21/27 (77.8%) for those currently taking corticosteroids. Fifteen patients had received rituximab within the 12 months preceding vaccination and 11/15 (73.3%) seroconverted in response to vaccine compared to 27/31 (87.1%) unexposed to treatment (*p* = 0.41; Fisher's exact test).

## Discussion

The data reveal lower seroconversion rates after one dose of a COVID-19 vaccination in a multi-ethnic group of patients living with AAV and renal involvement when compared to the general UK population, regardless of vaccination type. There was a trend to lower seroconversion rates in patients who had received Rituximab in the last 12 months and those who were currently receiving steroids, albeit significance thresholds were not met, likely because of small samples sizes. These observations complement other studies that reveal similarly impaired seroconversion rates in patients receiving immunomodulatory treatment, and add to the mounting evidence of reduced seroconversion in patients with renal disease from any cause (15). These data are the first to our knowledge to examine antibody responses to COVID-19 vaccination in patients under follow-up and treatment for AAV with renal involvement in a real-world setting.

The assay used in this audit has been validated both in published data and internally by the Clinical Transplantation Laboratory at this referral centre on a panel of SARS-CoV-2 positive samples and negative controls (12, 13). In published studies assessing seroconversion in response to vaccination, many different validated assays are used, including but not limited to assays which measure either the humoral or cellular response to COVID-19 antigens, and neutralisation assays (16). Differences seen in seroconversion rates between this audit and published studies may reflect inter-assay variability, different patient demographics, the prevalence of COVID-19 in the general population at the time of the study, as well as the number of patients in the study with prior COVID-19 infection. One limitation of our study is that we were unable to assess T-cell responses to vaccination due to service-provision constraints in our hospital. These constraints are likely to reflect the reality across some hospitals in the UK. These considerations illustrate that seroconversion to vaccination is just one surrogate marker of immunity to COVID-19, and further underscores the need for close links between hospital departments and their patients to acquire a holistic assessment of those at high-risk of COVID-19.

The COVID-19 pandemic has affected all communities around the world, and hospitals have had to respond differently to the specific needs of their local populations. Our hospital serves a particularly diverse population and consequently we were interested in whether seroconversion rates differed by ethnicity. Subgroup analyses show a trend to lower seroconversion in non-white patients after one dose, that resolved after two doses. It should be noted that conclusions drawn from subgroup analyses in this paper are caveated by small sample size. Despite these limitations, this report highlights the role of hospital-led identification and screening of especially vulnerable patients with complex disease in the local area. By identifying those patients who have not seroconverted to COVID-19 vaccination, we are able to fine-tune our advice to these patients as well as encourage further booster vaccinations in line with government guidance. In addition, this report demonstrates the utility of institutional flexibility to evaluate real-world trends in seroconversion. Through early identification of our AAV cohort, we were able to respond to the different timings of their first vaccination dose to produce a rolling picture of seroconversion rates in this group.

In the UK, at the time of writing, a high proportion of adults, and all clinically vulnerable adults, have been offered two doses of a COVID-19 vaccination. In addition, in November 2021, after the time-period captured by this study, the UK government recommended that all adults over 40 years are offered a booster dose 6 months after their second dose in part to mitigate waning immunity to COVID-19 in an at-risk cohort. There is very limited evidence on rates of COVID-19 infection after two doses (and even less after three doses) of vaccine in patients with renal disease although some studies have recently been published (17). In Grupper et al.'s study of patients undergoing haemodialysis, 2/56 developed COVID-19 after full vaccination whilst 4 patients developed COVID-19 requiring hospitalisation in a study of 308 renal transplant recipients (5, 18). Further large-scale studies of renal patients are needed in this area but until then, hospitals may have to develop a “watch-list” of particularly vulnerable patients that may be at higher risk of developing COVID-19 after vaccination. This audit highlights how departments might achieve meaningful systems to identify such patients.

This data corroborates previous reports of concurrent treatment with Rituximab impairing seroconversion rates in response to vaccination, although our data is not statistically significant, likely due to small subgroups (19). One limitation of this study is that we were unable to report B-cell depletion in response to Rituximab therapy, a likely explanation for the lower seroconversion trends seen in Rituximab-treated patients. Historic treatment with cyclophosphamide does not impair seroconversion, nor does current treatment with azathioprine or MMF albeit numbers were too small to draw firm conclusions. The seroconversion rate was lower in those currently taking steroids, than those who were not, however this data is likely to be confounded somewhat by the fact that 13/27 patients currently taking steroids were on Rituximab maintenance therapy. Indeed most patients currently on steroids but who had not received Rituximab in the last 12 months successfully seroconverted following two vaccine doses. It is important to note that most of our patients are taking multiple immunosuppressant medications, making it difficult to quantify and compare the relative effects of individual treatments on seroconversion rates. These regimens are typical of treatment of patients with AAV and an unavoidable complication of collecting real-world data from an AAV cohort of this size. Where conclusions can be drawn is when they corroborate published data. This report provides a real-world assessment of seroconversion rates in response to COVID-19 vaccination in a cohort of patients with AAV and renal involvement at a single centre. It highlights the importance of identifying the most vulnerable patients at the local community level. It provides some descriptive data to add to the growing evidence base highlighting the unique challenges of COVID-19 in nephrology patients.

## Data Availability Statement

The original contributions presented in the study are included in the article/supplementary material, further inquiries can be directed to the corresponding author/s.

## Author Contributions

JC and JW contributed data collection, data analysis, and report writing. AG, DK, AC, and NP contributed data analysis. MY contributed manuscript editing. RR contributed data collection, report writing, and manuscript editing. All authors contributed to the article and approved the submitted version.

## Conflict of Interest

The authors declare that the research was conducted in the absence of any commercial or financial relationships that could be construed as a potential conflict of interest.

## Publisher's Note

All claims expressed in this article are solely those of the authors and do not necessarily represent those of their affiliated organizations, or those of the publisher, the editors and the reviewers. Any product that may be evaluated in this article, or claim that may be made by its manufacturer, is not guaranteed or endorsed by the publisher.

## References

[1] Cattran DC Feehally J Cook HT Liu ZH Fervenza FC Mezzano SA. Kidney disease: Improving global outcomes (KDIGO) glomerulonephritis work group. KDIGO clinical practice guideline for glomerulonephritis. Kidney Int Suppl. (2012) 2:139–274. 10.1038/kisup.2012.923871408

[2] Polack FP Thomas SJ Kitchin N Absalon J Gurtman A Lockhart S. Safety and Efficacy of the BNT162b2 mRNA Covid-19 Vaccine. N Engl J Med. (2020) 383:2603–15. 10.1056/NEJMoa2034577 33301246 PMC7745181

[3] Voysey M Clemens SAC Madhi SA Weckx LY Folegatti PM Aley PK. Safety and efficacy of the ChAdOx1 nCoV-19 vaccine (AZD1222) against SARS-CoV-2: an interim analysis of four randomised controlled trials in Brazil, South Africa, and the UK. Lancet. (2021) 397:99–111. 10.1016/S0140-6736(20)32661-1 33306989 PMC7723445

[4] Wei J Stoesser N Matthews PC Studley R Bell I Bell JI. Antibody responses to SARS-CoV-2 vaccines in 45,965 adults from the general population of the United Kingdom. Nat Microbiol. (2021) 6:1140–9. 10.1101/2021.04.22.21255911 34290390 PMC8294260

[5] Grupper A Sharon N Finn T Cohen R Israel M Agbaria A. Humoral response to the Pfizer BNT162b2 vaccine in patients undergoing maintenance hemodialysis. Clin J Am Soc Nephrol. (2021) 16:1037–42. 10.2215/CJN.03500321 33824157 PMC8425628

[6] Stumpf J Siepmann T Lindner T Karger C Schwöbel J Anders L. The Lancet Regional Health - Europe Humoral and cellular immunity to SARS-CoV-2 vaccination in renal transplant versus dialysis patients : a prospective, multicenter observational study using mRNA-1273 or BNT162b2 mRNA vaccine. Lancet Reg Heal - Eur. (2021) 9:100178. 10.1016/j.lanepe.2021.100178 34318288 PMC8299287

[7] Bird S Panopoulou A Shea RL Tsui M Saso R Sud A. Response to first vaccination against SARS-CoV-2 in patients with multiple myeloma. Lancet Haematol. (2021) 3026:19–21. 10.1016/S2152-2650(21)02271-0 33887255 PMC8055205

[8] Mahil SK Bechman K Raharja A Domingo-vila C Baudry D Brown MA. Articles The effect of methotrexate and targeted immunosuppression on humoral and cellular immune responses to the COVID-19 vaccine BNT162b2 : a cohort study. Lancet Rheumatol. (2021) 9913:1–11. 10.26226/morressier.61081ff8bc981037240fe3c0 PMC8266273 34258590

[9] Bingham CO Looney RJ Deodhar A Halsey N Greenwald M Codding C. Immunization responses in rheumatoid arthritis patients treated with rituximab: Results from a controlled clinical trial. Arthritis Rheum. (2010) 62:64–74. 10.1002/art.25034 20039397

[10] Mrak D Tobudic S Koblischke M Graninger M Radner H Sieghart D. CoV-2 vaccination in rituximab- - treated patients : B cells promote humoral immune responses in the presence of T- - cell- - mediated immunity. Ann Rheum Dis. (2021) 80:1345–50. 10.1136/annrheumdis-2021-220781 34285048

[11] Haberman RH Herati RS Simon D Samanovic M Blank RB Tuen M. Methotrexate hampers immunogenicity to BNT162B2 mRNA covid-19 vaccine in immune-mediated inflammatory disease. Ann Rheum Dis. (2020) 1–6. 10.1101/2021.05.11.21256917 34035003 PMC8219484

[12] Becker M Strengert M Junker D Kaiser PD Kerrinnes T Traenkle B. Exploring beyond clinical routine SARS-CoV-2 serology using MultiCoV-Ab to evaluate endemic coronavirus cross-reactivity. Nat Commun. (2021) 12:1–12. 10.1038/s41467-021-20973-3 33608538 PMC7896075

[13] Becker M Dulovic A Junker D Ruetalo N Kaiser PD Pinilla YT. Immune response to SARS-CoV-2 variants of concern in vaccinated individuals. Nat Commun. (2021) 12:1–8. 10.1038/s41467-021-23473-6 34035301 PMC8149389

[14] Juraszek J Rutten L Blokland S Bouchier P Voorzaat R Ritschel T. Stabilizing the closed SARS-CoV-2 spike trimer. Nat Commun. (2021) 12:1–8. 10.1038/s41467-020-20321-x 33431842 PMC7801441

[15] Carr EJ Kronbichler A Graham-Brown M Abra G Argyropoulos C Harper L. Systematic review of early immune response to SARS-CoV-2 vaccination among patients with chronic kidney disease. Kidney Int reports. (2021) 6:2292–304. 10.1016/j.ekir.2021.06.027 34250319 PMC8257418

[16] Ikizler TA Coates PT Rovin BH. Immune response to SARS-CoV-2 infection and vaccination in patients receiving kidney replacement therapy. Kidney Int. (2021) 99:1275–9. 10.1016/j.kint.2021.04.007 33931226 PMC8055920

[17] Forbes S Davari M Gnanasampanthan S Roth N Young G Rajakariar R. Persistence of antibody response to SARS-CoV-2 in a cohort of haemodialysis patients with COVID-19. Nephrol Dial Transplant. (2021) 36:1292–7. 10.1093/ndt/gfab066 33720379 PMC7989221

[18] Rozen-Zvi B Yahav D Agur T Zingerman B Ben-Zvi H Atamna A. Antibody response to SARS-CoV-2 mRNA vaccine among kidney transplant recipients: a prospective cohort study. Clin Microbiol Infect. (2021) 27:4–7. 10.1016/j.cmi.2021.04.028 33957273 PMC8091803

[19] Prendecki M Clarke C Edwards H McIntyre S Mortimer P Gleeson S. Humoral and T-cell responses to SARS-CoV-2 vaccination in patients receiving immunosuppression. Ann Rheum Dis. (2021) 80:1322–1329. 10.1136/annrheumdis-2021-220626 34362747 PMC8350975

